# Supplementary material for: A Likelihood Approach for Real-Time Calibration of Stochastic Compartmental Epidemic Models
Source: PLoS Comput Biol. 2017 Jan 17;13(1):e1005257. doi: 10.1371/journal.pcbi.1005257 (PMC5240920; doi:10.1371/journal.pcbi.1005257)
Supplement: S1 File — (TAR.GZ) [file pcbi.1005257.s014.tar.gz › HSPH_Online-SI-Revision/PF08/n1-extreme/n1-extreme_PF2_table-8.pdf]

1	newly 3 weeks spec	6.	62.28	{4., 151.}	955.	0.917056	newly 3 weeks cumul	8.	166.97	{11., 426.}	1997.38	1.1012	newly 5 weeks spec	31.	56.71	{2., 119.}	126.355	0.544063	newly 5 weeks cumul	58.	281.9	{25., 621.}	409.207	0.697037	newly 68 weeks cumul	923.	614.4	{176., 861.}	33.4518	0.377601
2	newly 3 weeks spec	47.	48.67	{3., 103.}	67.4255	0.461929	newly 3 weeks cumul	87.	139.75	{14., 290.}	106.264	0.41773	newly 5 weeks spec	79.	40.72	{4., 87.}	53.6456	0.559705	newly 5 weeks cumul	238.	226.01	{28., 435.}	58.5	0.387866	newly 64 weeks cumul	911.	465.29	{61., 803.}	48.9407	0.438917
3	newly 3 weeks spec	17.	46.26	{2., 104.}	208.471	0.585671	newly 3 weeks cumul	33.	125.49	{8., 312.}	309.909	0.603024	newly 5 weeks spec	43.	49.39	{2., 92.}	71.0465	0.47431	newly 5 weeks cumul	103.	223.53	{17., 491.}	156.165	0.512823	newly 66 weeks cumul	907.	566.71	{80., 842.}	37.5667	0.402726
4	newly 3 weeks spec	11.	45.56	{6., 98.}	335.455	0.620783	newly 3 weeks cumul	27.	121.75	{14., 268.}	371.444	0.633822	newly 5 weeks spec	51.	45.49	{4., 88.}	59.549	0.464571	newly 5 weeks cumul	107.	214.73	{27., 447.}	137.187	0.450396	newly 65 weeks cumul	788.	545.09	{102., 828.}	33.2627	0.326787
5	newly 3 weeks spec	48.	37.86	{12., 81.}	50.9167	0.342651	newly 3 weeks cumul	91.	118.84	{43., 246.}	64.1099	0.232592	newly 5 weeks spec	68.	31.38	{5., 64.}	57.4706	0.540093	newly 5 weeks cumul	214.	184.87	{76., 389.}	49.2196	0.271204	newly 58 weeks cumul	910.	394.51	{103., 688.}	56.6473	0.472788
6	newly 3 weeks spec	34.	45.84	{3., 90.}	85.5294	0.465943	newly 3 weeks cumul	108.	142.13	{18., 277.}	80.8981	0.39688	newly 5 weeks spec	27.	36.84	{2., 76.}	90.6667	0.497121	newly 5 weeks cumul	166.	221.51	{31., 412.}	80.0422	0.392882	newly 63 weeks cumul	696.	436.49	{77., 716.}	40.6853	0.373979
7	newly 3 weeks spec	32.	60.44	{1., 135.}	139.188	0.612081	newly 3 weeks cumul	67.	160.21	{4., 388.}	183.358	0.607316	newly 5 weeks spec	59.	54.9	{0., 102.}	59.5932	0.596593	newly 5 weeks cumul	173.	275.75	{4., 600.}	110.746	0.591301	newly 67 weeks cumul	867.	595.66	{4., 884.}	32.1615	0.4811
8	newly 3 weeks spec	33.	35.87	{1., 68.}	71.3636	0.535757	newly 3 weeks cumul	77.	109.85	{5., 209.}	93.5974	0.488843	newly 5 weeks spec	70.	29.68	{0., 60.}	59.7143	0.720485	newly 5 weeks cumul	203.	173.12	{8., 332.}	54.4532	0.473589	newly 61 weeks cumul	775.	379.92	{45., 661.}	51.6232	0.520844
9	newly 3 weeks spec	10.	62.45	{6., 145.}	544.5	0.746531	newly 3 weeks cumul	20.	163.58	{11., 391.}	733.9	0.773344	newly 5 weeks spec	41.	59.65	{5., 123.}	97.2439	0.513126	newly 5 weeks cumul	80.	283.84	{27., 609.}	281.45	0.594395	newly 68 weeks cumul	770.	627.09	{196., 867.}	25.5857	0.285387
10	newly 3 weeks spec	30.	31.43	{2., 67.}	71.7	0.512	newly 3 weeks cumul	68.	97.19	{5., 205.}	96.0147	0.458137	newly 5 weeks spec	62.	25.36	{1., 54.}	61.6774	0.723818	newly 5 weeks cumul	180.	150.81	{16., 305.}	57.8944	0.451021	newly 61 weeks cumul	869.	349.96	{45., 653.}	59.8596	0.596677
11	newly 3 weeks spec	30.	60.44	{1., 135.}	150.133	0.619093	newly 3 weeks cumul	41.	160.21	{4., 388.}	324.902	0.693922	newly 5 weeks spec	78.	54.9	{0., 102.}	50.1538	0.621263	newly 5 weeks cumul	165.	275.75	{4., 600.}	117.376	0.596647	newly 67 weeks cumul	767.	595.66	{4., 884.}	29.3977	0.452883
12	newly 3 weeks spec	6.	62.28	{4., 151.}	955.	0.917056	newly 3 weeks cumul	12.	166.97	{11., 426.}	1304.25	0.956275	newly 5 weeks spec	33.	56.71	{2., 119.}	117.242	0.537546	newly 5 weeks cumul	59.	281.9	{25., 621.}	401.254	0.692583	newly 68 weeks cumul	724.	614.4	{176., 861.}	26.5525	0.317664
13	newly 3 weeks spec	17.	31.73	{1., 67.}	134.882	0.565554	newly 3 weeks cumul	47.	98.15	{5., 201.}	151.043	0.518898	newly 5 weeks spec	37.	26.61	{2., 57.}	55.2703	0.537691	newly 5 weeks cumul	107.	153.63	{15., 297.}	94.0654	0.45025	newly 61 weeks cumul	729.	366.85	{57., 661.}	50.5528	0.473162
14	newly 3 weeks spec	25.	45.3	{5., 97.}	127.28	0.50455	newly 3 weeks cumul	67.	125.3	{13., 271.}	130.507	0.480576	newly 5 weeks spec	83.	43.5	{3., 88.}	52.	0.5831	newly 5 weeks cumul	206.	213.87	{18., 431.}	66.8204	0.450096	newly 65 weeks cumul	782.	531.08	{78., 825.}	34.3581	0.368743
15	newly 3 weeks spec	26.	45.99	{14., 86.}	97.5769	0.28627	newly 3 weeks cumul	51.	136.07	{46., 254.}	171.235	0.363714	newly 5 weeks spec	70.	39.81	{15., 69.}	46.5857	0.345962	newly 5 weeks cumul	147.	217.96	{78., 400.}	70.068	0.225954	newly 60 weeks cumul	692.	477.62	{278., 751.}	34.2919	0.212884
16	newly 3 weeks spec	24.	59.23	{1., 128.}	188.792	0.633235	newly 3 weeks cumul	40.	157.05	{4., 388.}	325.275	0.68501	newly 5 weeks spec	42.	53.64	{0., 102.}	80.9048	0.590325	newly 5 weeks cumul	120.	269.35	{4., 596.}	166.275	0.623096	newly 67 weeks cumul	755.	592.43	{4., 874.}	28.894	0.444965
17	newly 3 weeks spec	26.	47.73	{3., 104.}	128.808	0.519583	newly 3 weeks cumul	47.	138.28	{14., 278.}	222.043	0.516615	newly 5 weeks spec	50.	40.62	{4., 86.}	54.52	0.471509	newly 5 weeks cumul	121.	223.23	{27., 435.}	123.793	0.434491	newly 64 weeks cumul	819.	466.02	{61., 783.}	43.6923	0.402369
18	newly 3 weeks spec	13.	59.32	{1., 137.}	386.769	0.752546	newly 3 weeks cumul	23.	155.5	{4., 400.}	600.087	0.819925	newly 5 weeks spec	42.	54.21	{0., 106.}	84.5952	0.60468	newly 5 weeks cumul	99.	268.17	{4., 609.}	207.081	0.648431	newly 67 weeks cumul	912.	583.47	{4., 884.}	36.0471	0.513632
19	newly 3 weeks spec	14.	62.16	{4., 151.}	373.143	0.678812	newly 3 weeks cumul	34.	166.67	{11., 426.}	417.618	0.65065	newly 5 weeks spec	39.	56.16	{2., 119.}	96.5641	0.528371	newly 5 weeks cumul	99.	280.68	{25., 621.}	218.707	0.579605	newly 68 weeks cumul	880.	613.15	{176., 861.}	30.6875	0.359284
20	newly 3 weeks spec	50.	34.36	{1., 68.}	52.76	0.556165	newly 3 weeks cumul	121.	104.95	{5., 205.}	56.6198	0.470626	newly 5 weeks spec	81.	28.44	{3., 60.}	65.4568	0.741579	newly 5 weeks cumul	273.	165.55	{10., 321.}	50.2674	0.502501	newly 61 weeks cumul	893.	383.59	{74., 661.}	57.1187	0.52926
21	newly 3 weeks spec	10.	47.68	{3., 103.}	400.2	0.692067	newly 3 weeks cumul	39.	137.06	{14., 290.}	276.256	0.557643	newly 5 weeks spec	27.	40.17	{3., 87.}	99.1481	0.485546	newly 5 weeks cumul	84.	222.12	{27., 435.}	193.762	0.497755	newly 64 weeks cumul	711.	459.03	{60., 803.}	40.2489	0.379358
22	newly 3 weeks spec	50.	42.06	{2., 92.}	62.96	0.522593	newly 3 weeks cumul	92.	115.52	{12., 259.}	81.9783	0.456152	newly 5 weeks spec	80.	42.02	{3., 85.}	52.95	0.581045	newly 5 weeks cumul	222.	200.52	{18., 424.}	60.2072	0.456392	newly 65 weeks cumul	825.	520.51	{84., 825.}	37.6448	0.392205
23	newly 3 weeks spec	21.	60.3	{1., 135.}	225.714	0.660962	newly 3 weeks cumul	38.	159.83	{4., 388.}	352.447	0.705374	newly 5 weeks spec	50.	54.93	{0., 102.}	67.5	0.581765	newly 5 weeks cumul	115.	274.84	{4., 600.}	178.817	0.633848	newly 67 weeks cumul	744.	599.92	{4., 876.}	28.6048	0.440071
24	newly 3 weeks spec	11.	62.28	{4., 151.}	489.818	0.735309	newly 3 weeks cumul	16.	166.97	{11., 426.}	959.188	0.861104	newly 5 weeks spec	41.	56.71	{2., 119.}	91.3415	0.522721	newly 5 weeks cumul	85.	281.9	{25., 621.}	262.329	0.608146	newly 68 weeks cumul	886.	614.4	{176., 861.}	30.921	0.360896
25	newly 3 weeks spec	21.	43.99	{6., 97.}	148.524	0.502712	newly 3 weeks cumul	34.	117.43	{13., 259.}	271.324	0.573957	newly 5 weeks spec	31.	44.36	{3., 88.}	95.871	0.469181	newly 5 weeks cumul	93.	207.6	{26., 435.}	156.43	0.469973	newly 65 weeks cumul	758.	534.04	{102., 828.}	33.7995	0.326238
26	newly 3 weeks spec	12.	60.76	{1., 135.}	435.333	0.785767	newly 3 weeks cumul	15.	160.94	{4., 388.}	994.133	0.964403	newly 5 weeks spec	48.	55.4	{0., 102.}	71.625	0.586107	newly 5 weeks cumul	84.	277.09	{4., 600.}	262.726	0.695439	newly 67 weeks cumul	923.	600.96	{4., 883.}	34.8906	0.495875
27	newly 3 weeks spec	38.	37.68	{2., 77.}	62.6842	0.493615	newly 3 weeks cumul	98.	110.94	{5., 231.}	70.3265	0.480022	newly 5 weeks spec	56.	35.86	{0., 77.}	54.3214	0.616645	newly 5 weeks cumul	192.	183.57	{7., 355.}	58.7865	0.495348	newly 62 weeks cumul	795.	422.73	{18., 776.}	48.5673	0.585029
28	newly 3 weeks spec	39.	48.52	{2., 104.}	84.1538	0.515434	newly 3 weeks cumul	74.	131.28	{7., 302.}	126.081	0.492878	newly 5 weeks spec	88.	50.95	{2., 92.}	50.5114	0.568321	newly 5 weeks cumul	201.	233.02	{11., 491.}	73.6318	0.461601	newly 66 weeks cumul	916.	569.78	{34., 842.}	37.8253	0.415985
29	newly 3 weeks spec	8.	45.38	{4., 91.}	488.25	0.746956	newly 3 weeks cumul	18.	131.66	{13., 263.}	646.444	0.780361	newly 5 weeks spec	61.	39.46	{4., 80.}	51.5738	0.503388	newly 5 weeks cumul	116.	213.84	{25., 406.}	124.983	0.452118	newly 63 weeks cumul	930.	463.01	{61., 781.}	50.229	0.464788
30	newly 3 weeks spec	51.	39.46	{14., 73.}	46.0392	0.272966	newly 3 weeks cumul	155.	122.02	{45., 224.}	44.6452	0.256115	newly 5 weeks spec	44.	32.94	{9., 63.}	50.	0.350911	newly 5 weeks cumul	251.	191.55	{75., 353.}	44.5139	0.261602	newly 58 weeks cumul	605.	384.72	{128., 658.}	42.2545	0.298934
31	newly 3 weeks spec	24.	60.76	{1., 135.}	194.833	0.643984	newly 3 weeks cumul	52.	160.94	{4., 388.}	246.692	0.644935	newly 5 weeks spec	60.	55.4	{0., 102.}	57.9	0.588637	newly 5 weeks cumul	138.	277.09	{4., 600.}	145.138	0.612196	newly 67 weeks cumul	874.	600.96	{4., 883.}	31.7872	0.474522
32	newly 3 weeks spec	21.	59.32	{1., 137.}	222.	0.655693	newly 3 weeks cumul	42.	155.5	{4., 400.}	303.19	0.67119	newly 5 weeks spec	41.	54.21	{0., 106.}	86.9512	0.605936	newly 5 weeks cumul	113.	268.17	{4., 609.}	177.177	0.628725	newly 67 weeks cumul	845.	583.47	{4., 884.}	32.7041	0.48779
33	newly 3 weeks spec	14.	42.26	{3., 85.}	230.714	0.596125	newly 3 weeks cumul	41.	124.	{11., 249.}	230.293	0.545706	newly 5 weeks spec	35.	38.09	{3., 79.}	72.7143	0.470942	newly 5 weeks cumul	96.	202.6	{19., 401.}	146.479	0.476317	newly 63 weeks cumul	860.	443.21	{61., 761.}	48.643	0.467666
34	newly 3 weeks spec	20.	45.67	{2., 102.}	169.15	0.558956	newly 3 weeks cumul	48.	123.59	{8., 301.}	195.938	0.530321	newly 5 weeks spec	70.	48.96	{2., 92.}	52.0857	0.521673	newly 5 weeks cumul	151.	220.57	{17., 480.}	96.7086	0.467918	newly 66 weeks cumul	845.	563.87	{80., 842.}	34.0462	0.378292
35	newly 3 weeks spec	18.	60.13	{1., 128.}	269.833	0.693369	newly 3 weeks cumul	50.	159.06	{4., 388.}	256.48	0.65678	newly 5 weeks spec	58.	54.84	{0., 102.}	59.6897	0.592866	newly 5 weeks cumul	143.	273.59	{4., 596.}	138.147	0.611583	newly 67 weeks cumul	834.	586.87	{4., 876.}	31.5144	0.480609
36	newly 3 weeks spec	24.	46.66	{3., 103.}	139.5	0.529787	newly 3 weeks cumul	59.	132.71	{14., 282.}	161.	0.465688	newly 5 weeks spec	53.	40.54	{4., 87.}	54.7547	0.48935	newly 5 weeks cumul	146.	217.51	{27., 435.}	95.5274	0.408632	newly 64 weeks cumul	848.	470.9	{61., 805.}	44.7807	0.412113
37	newly 3 weeks spec	45.	37.08	{14., 68.}	43.3778	0.285323	newly 3 weeks cumul	72.	111.15	{41., 193.}	73.4583	0.230754	newly 5 weeks spec	59.	34.15	{13., 62.}	51.3051	0.420656	newly 5 weeks cumul	179.	182.28	{76., 335.}	44.1676	0.208483	newly 59 weeks cumul	848.	425.99	{198., 692.}	50.0767	0.373762
38	newly 3 weeks spec	27.	46.6	{3., 103.}	122.074	0.512808	newly 3 weeks cumul	70.	133.38	{14., 290.}	133.029	0.451812	newly 5 weeks spec	62.	39.5	{3., 87.}	53.3871	0.537294	newly 5 weeks cumul	186.	216.95	{27., 435.}	74.2097	0.412467	newly 64 weeks cumul	878.	453.37	{60., 803.}	48.4795	0.460855
39	newly 3 weeks spec	9.	60.13	{1., 128.}	595.444	0.864492	newly 3 weeks cumul	17.	159.06	{4., 388.}	859.412	0.922109	newly 5 weeks spec	28.	54.84	{0., 102.}	138.429	0.646022	newly 5 weeks cumul	66.	273.59	{4., 596.}	345.591	0.752972	newly 67 weeks cumul	893.	586.87	{4., 876.}	34.3908	0.502844
40	newly 3 weeks spec	58.	43.6	{5., 96.}	58.931	0.523354	newly 3 weeks cumul	138.	118.35	{13., 268.}	61.9058	0.478076	newly 5 weeks spec	45.	42.77	{3., 88.}	61.8444	0.489825	newly 5 weeks cumul	245.	205.3	{18., 424.}	57.5592	0.463691	newly 65 weeks cumul	775.	524.67	{78., 825.}	34.8994	0.372773
41	newly 3 weeks spec	31.	25.65	{1., 63.}	63.5806	0.550584	newly 3 weeks cumul	62.	85.45	{7., 183.}	94.1774	0.442362	newly 5 weeks spec	37.	20.61	{0., 45.}	58.027	0.670555	newly 5 weeks cumul	130.	129.75	{9., 285.}	67.2538	0.439648	newly 60 weeks cumul	655.	294.88	{20., 586.}	57.5969	0.59335
42	newly 3 weeks spec	20.	62.28	{4., 151.}	247.3	0.605303	newly 3 weeks cumul	35.	166.97	{11., 426.}	404.257	0.64352	newly 5 weeks spec	52.	56.71	{2., 119.}	72.6346	0.525421	newly 5 weeks cumul	108.	281.9	{25., 621.}	196.889	0.561232	newly 68 weeks cumul	918.	614.4	{176., 861.}	33.1002	0.375289
43	newly 3 weeks spec	9.	59.23	{1., 128.}	584.333	0.851812	newly 3 weeks cumul	17.	157.05	{4., 388.}	846.059	0.911958	newly 5 weeks spec	64.	53.64	{0., 102.}	54.3438	0.594629	newly 5 weeks cumul	106.	269.35	{4., 596.}	192.613	0.640968	newly 67 weeks cumul	902.	592.43	{4., 874.}	34.3891	0.492655
44	newly 3 weeks spec	40.	44.32	{6., 97.}	76.2	0.483284	newly 3 weeks cumul	67.	119.67	{13., 259.}	122.104	0.45537	newly 5 weeks spec	68.	44.44	{3., 88.}	53.8235	0.514179	newly 5 weeks cumul	179.	209.79	{26., 436.}	75.0447	0.422094	newly 65 weeks cumul	873.	539.87	{102., 828.}	38.2761	0.363431
45	newly 3 weeks spec	11.	62.16	{4., 151.}	489.455	0.734898	newly 3 weeks cumul	17.	166.67	{11., 426.}	896.882	0.83882	newly 5 weeks spec	50.	56.16	{2., 119.}	75.72	0.529908	newly 5 weeks cumul	90.	280.68	{25., 621.}	244.844	0.597274	newly 68 weeks cumul	926.	613.15	{176., 861.}	33.7959	0.379908
46	newly 3 weeks spec	19.	45.25	{6., 98.}	172.579	0.502349	newly 3 weeks cumul	34.	120.94	{14., 268.}	279.882	0.568966	newly 5 weeks spec	55.	45.01	{4., 88.}	56.6	0.471627	newly 5 weeks cumul	129.	212.87	{27., 447.}	108.256	0.427257	newly 65 weeks cumul	889.	541.26	{102., 828.}	39.1564	0.372441
47	newly 3 weeks spec	19.	46.62	{2., 104.}	184.737	0.570877	newly 3 weeks cumul	29.	126.24	{8., 312.}	362.414	0.634258	newly 5 weeks spec	39.	49.56	{2., 92.}	79.0769	0.476616	newly 5 weeks cumul	94.	224.54	{17., 491.}	175.596	0.528393	newly 66 weeks cumul	840.	566.37	{80., 842.}	33.4821	0.373192
48	newly 3 weeks spec	17.	50.06	{2., 116.}	227.412	0.58738	newly 3 weeks cumul	48.	139.39	{8., 332.}	225.688	0.554619	newly 5 weeks spec	41.	51.09	{2., 93.}	74.561	0.463713	newly 5 weeks cumul	126.	241.8	{17., 530.}	132.794	0.493678	newly 66 weeks cumul	757.	576.37	{80., 841.}	29.4042	0.335107
49	newly 3 weeks spec	30.	62.16	{4., 151.}	155.2	0.555242	newly 3 weeks cumul	64.	166.67	{11., 426.}	204.359	0.545057	newly 5 weeks spec	69.	56.16	{2., 119.}	60.6957	0.558787	newly 5 weeks cumul	166.	280.68	{25., 621.}	118.06	0.508517	newly 68 weeks cumul	950.	613.15	{176., 861.}	35.4579	0.390974
50	newly 3 weeks spec	39.	56.27	{4., 119.}	95.4103	0.480825	newly 3 weeks cumul	92.	160.05	{14., 360.}	119.924	0.464741	newly 5 weeks spec	56.	49.85	{2., 93.}	52.125	0.470493	newly 5 weeks cumul	194.	262.85	{19., 530.}	84.3041	0.43544	newly 66 weeks cumul	717.	563.84	{34., 840.}	30.8954	0.336019
